# Unraveling the ecology and epidemiology of an emerging fungal disease, sea turtle egg fusariosis (STEF)

**DOI:** 10.1371/journal.ppat.1007682

**Published:** 2019-05-16

**Authors:** Christopher W. Smyth, Jullie M. Sarmiento-Ramírez, Dylan P. G. Short, Javier Diéguez-Uribeondo, Kerry O’Donnell, David M. Geiser

**Affiliations:** 1 Department of Plant Pathology and Environmental Microbiology, Pennsylvania State University, University Park, Pennsylvania, United States of America; 2 Departamento de Micología, Real Jardín Botánico-CSIC, Madrid, Spain; 3 Department of Plant Pathology, University of California-Davis, Salinas, California, United States of America; 4 Mycotoxin Prevention and Applied Microbiology Research Unit, National Center for Agricultural Utilization Research, United States Department of Agriculture, Agricultural Research Service, Peoria, Illinois, United States of America; Geisel School of Medicine at Dartmouth, UNITED STATES

## Introduction

Emerging fungal diseases of wildlife are increasingly common, with far-reaching consequences for biodiversity and ecosystem health [1]. These include white-nose syndrome of bats in North America, which has killed over 5.5 million bats in the last decade [2], and chytridiomycosis, which has led to the global decline or extinction of at least 200 frog species [3]. Sea turtle egg fusariosis (STEF) is a newly emergent fungal disease linked to egg mortality in endangered sea turtle nests worldwide [4,5]. Two closely related fungal species, *Fusarium keratoplasticum* (*Fk*) and *F*. *falciforme* (*Ff*), have been implicated as the causative agents of STEF [4], but questions remain regarding the etiology and epidemiology of these pathogens. Primarily, it is unclear whether *Fk* and *Ff* are invasive pathogens or natural nest inhabitants causing disease under changing environmental conditions.

Effective management strategies for the control of emerging fungal diseases such as STEF require an understanding of whether a pathogen is novel or endemic [6]. A novel pathogen gains access to and infects naïve hosts as a result of migration of the pathogen or the development of novel pathogenic genotypes. Thus, effective management strategies must aim primarily at preventing pathogen introduction and expansion, often with a focus on potential disease vectors or other means of pathogen transmission. In contrast, endemic pathogens naturally occur in the host’s environment, but shifts in environmental conditions and/or host susceptibility heavily influence pathogenicity. Hence, management of disease caused by endemic pathogens relies on an understanding of environmental and host factors that influence disease emergence and severity. Because of these fundamental differences in management strategies, defining a pathogen as novel or endemic is a key first step toward mitigating disease impact on host populations [6].

Determining whether *Fk* and *Ff* are novel or endemic agents of STEF first requires an understanding of their ecology and distribution. This review focuses on the known ecological and epidemiological connections between *Fk* and *Ff* as human pathogens and inhabitants of the built environment and their recent emergence in association with STEF. Emphasis is placed herein on the importance of investigating these connections within a population biology framework to assess the endemic or novel nature of these pathogens for management purposes.

### What do we know about the *Fusarium* pathogens *Fk* and *Ff*?

**Fig 1:**
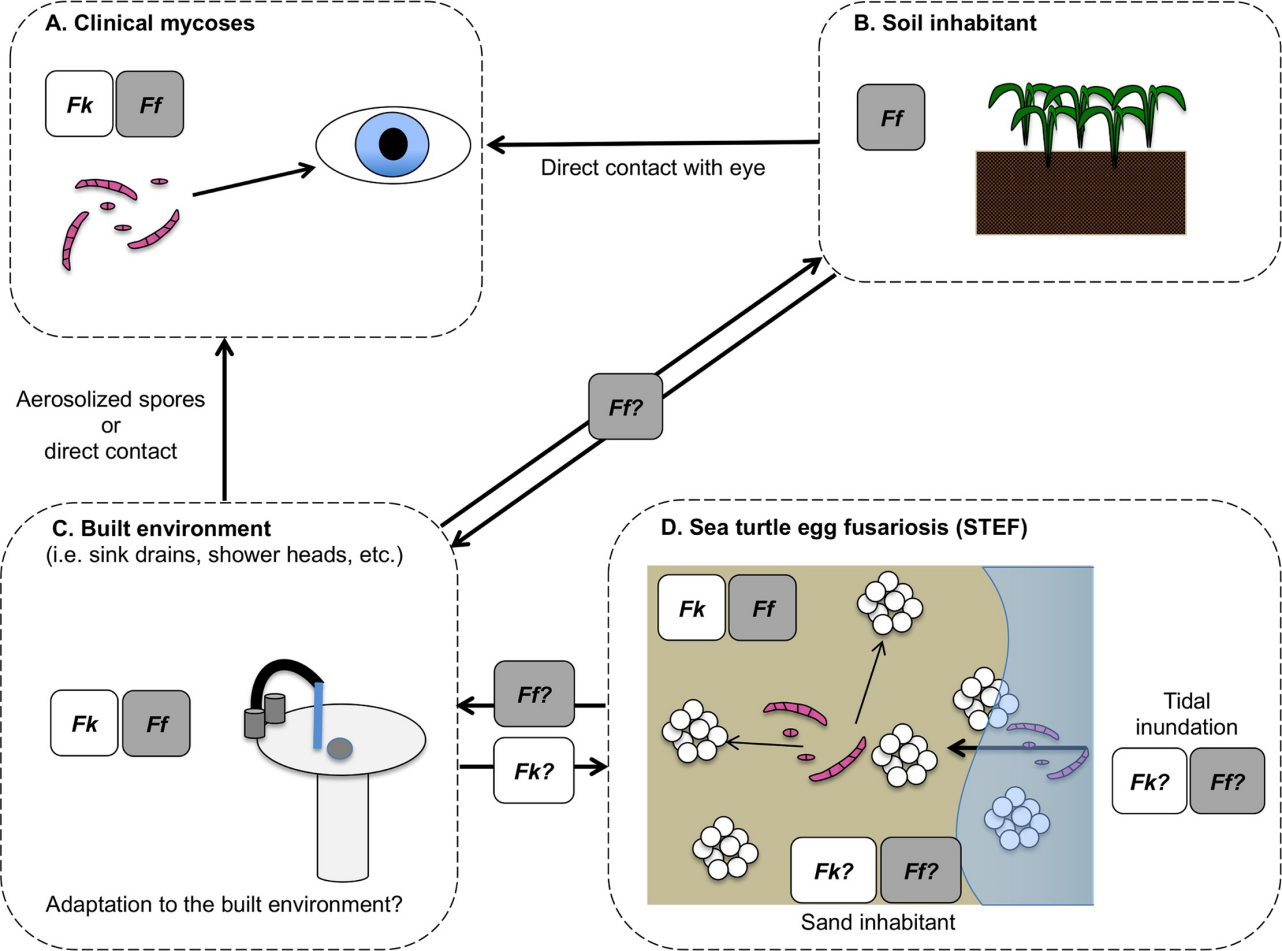
Infection model for *Fk* and *Ff*. (A) *Fk* and *Ff* are both associated with clinical infections, especially keratitis of the eye. (B) *Ff* is a common soil inhabitant, which is the reservoir implicated in corneal infections caused by this species. (C) *Fk* and *Ff* have both been found in the built environment, but *Fk* is typically present in much larger numbers. (D) *Fk* and *Ff* have both been found in association with sea turtle egg fusariosis. However, the epidemiology of these species as disease agents, and their potential connection to the built environment and human infections, is unknown. *Ff*, *F*. *falciforme*; *Fk*, *F*. *keratoplasticum*.

*Fusarium* is a diverse genus of ascomycete fungi, currently containing approximately 300 phylogenetic species distributed in 23 monophyletic lineages referred to as species complexes [7,8]. Fusaria are best known for their economic impacts as plant pathogens, such as Panama disease of bananas caused by *F*. *oxysporum* f.sp. *cubense* race 4 [9]. They are also well known for the public health impacts associated with production of mycotoxins such as trichothecenes, zearalenone, and fumonisins [10]. In addition, phylogenetically diverse *Fusarium* species are responsible for mycotic infections in humans and other animals. Approximately 75% of fusarioses are caused by members of one lineage, the *F*. *solani* species complex (FSSC) [11]. *Fk* and *Ff*, the two species known to be associated with STEF, are common, cosmopolitan members of the FSSC. They can cause life-threatening mycoses in immunocompromised or immunosuppressed humans, as well as cutaneous and subcutaneous infections, with a particular propensity to cause corneal infections in healthy individuals (Fig 1A) [12]. Outside of these infections, *Ff* is found around the world mostly as a soil-associated species (Fig 1B); however, prior to its discovery in association with STEF, *Fk* had only been isolated from nonsoil sources of high anthropogenic influence, particularly plumbing systems (Fig 1C) [13,14].

Anaissie and colleagues [15] formally proposed a link between nosocomial *Fusarium* infections and hospital water systems, finding evidence that these opportunistic fungi reside in certain parts of the hospital environment, such as showerheads and sink drains. Molecular evidence later reinforced these findings [11,16]. *Fk*, initially referred to as FSSC 2 [11], was later identified as one of the two most common species in the 2005 and 2006 outbreaks of fungal keratitis in contact lens users in Southeast Asia and North America [17]. A subsequent study, explicitly looking at *Fusarium* diversity in bathroom sink drains across the eastern United States, found *Fk* to be a dominant species, but *Ff* was also present in low levels [13], further suggesting that plumbing biofilms may serve as a reservoir for these opportunistic pathogens. Outside of these outbreaks associated with contact lens use, most *Ff* keratitis infections have been attributed to traumatic introduction of soil particles or plant debris into the eye, consistent with this species’ widespread association with soil environments [11].

### What is STEF?

*Fusarium* species have been isolated from the eggshells and embryonic tissue of failed sea turtle eggs for several decades, with hypotheses concerning their ecological role ranging from decomposition to pathogenicity [18]. Previous studies on the mycobiota from failed sea turtle eggs have reported a number of species, including members of the FSSC and *F*. *oxysporum* species complex [19]. Artificial incubation of sea turtle eggs with sand from nesting beaches suggested that once fungal invaders have colonized a failed egg, they are able to spread to adjacent healthy eggs and cause mortality [18]. In 2010, *Fk* and *Ff*, originally identified as *F*. *solani*, were associated with mass mortalities in the nests of loggerhead sea turtles (*Caretta caretta*) in Boa Vista, Cape Verde [5]. Koch’s postulates conducted with a strain of *Fk* isolated from eggs in Boa Vista provided evidence supporting *Fk* as a causative agent of STEF [5]. Koch’s postulates have not been conducted with *Ff*. Following this discovery, a worldwide survey of sea turtle nesting sites revealed that *Fk* and *Ff* were isolated from infected eggs from six of seven sea turtle species at major nesting sites in the Atlantic, Indian, and Pacific Oceans, as well as the Caribbean Sea [4,20]. Despite their global distribution in sea turtle nests, little is known about local and regional impacts of *Fk* and *Ff* on nesting sites and the factors that play a role in disease development.

Symptoms indicating that sea turtle eggs might be infected with *Fusarium* include the presence of atypically colored areas (e.g., yellow, blue, gray, red) on the eggshell, with more severe infections showing gray hyphal mats on the outside and the inside of the eggs and on the embryos [5]. However, *Fk* and *Ff* have also been isolated from asymptomatic eggs, suggesting that the presence of the pathogen may not be sufficient for disease to occur. Factors such as changing environmental conditions have been shown to influence hatching success and may also influence disease development [4]. Results of the aforementioned study also documented that disease incidence and nest mortality were significantly higher in nests exposed to tidal inundation or in sand with silt/clay composition compared with dry sand nests. High levels of clay and silt on nesting beaches have been shown to severely impact hatching success, particularly for loggerhead sea turtles, independent of disease [21].

There is still much we do not know about STEF, including the source(s) of *Fk* and *Ff*, as highlighted in Fig 1D, as well as environmental factors that may contribute to disease development. Are *Fk* and *Ff* native to sandy beach environments? Is their association with inundated nests indicative of a marine or sand-borne origin? Or are these pathogens introduced into the nest environment via runoff from the built environment, where they are associated with plumbing systems and opportunistic human infections? Distinguishing among these potential sources has important implications regarding the epidemiology and etiology of STEF and for formulating informed approaches focusing on prevention and management. The known global distribution of these opportunistic pathogens, in addition to the role environmental conditions play in disease incidence and severity, suggest an endemic nature, but more evidence is needed to support this hypothesis. Future research on this topic should include studies testing the association of *Fk* and *Ff* presence with hatch success in sea turtle nests, as well as experiments to determine the influence of environmental factors on *Fusarium* pathogenicity on sea turtle eggs.

### How can population genetics help unravel the ecology and epidemiology of the STEF pathogens?

Population genetic data provide a useful means for differentiating source and migrant populations of an emerging pathogen. Multilocus sequence-based genotyping has been used to determine the novel or endemic nature of fungal diseases, including white-nose syndrome [22], chytridiomycosis [6], and *Fusarium* diseases of humans [14]. The latter study significantly advanced our understanding of pathogen identity, genetic diversity, and clonal versus recombinant modes of reproduction and enabled key epidemiological inferences about populations of the opportunistic human pathogen *Fk*. By analyzing *Fusarium* populations from sea turtle eggs and nesting environments in the context of these studies, this method could be used to relate STEF to the known diversity of *Fk*, which is based mostly on human clinical and plumbing environments, and *Ff*, which is based mostly on human clinical and soil environments.

Short and colleagues [14] used a 9-locus sequence-based genotyping system that revealed very high levels of genetic diversity among a large collection of *Fk* isolates (n = 231) from human infections and other sources, with the majority isolated from plumbing environments. This analysis also revealed that the *Fk* 2-d sequence type was prevalent in human infections and sink drains. *Fk* 2-d was one of the two most common sequence types recovered from the 2005 and 2006 contact lens–associated outbreaks of mycotic keratitis [17]. On top of this strongly clonal pattern, evidence for historical recombination was discovered in *Fk* 2-d across a diverse array of genotypes. These results suggest a mixed reproductive model, involving sexual and asexual reproduction, allowing for adaptation to a changing environment and rapid spread of successful genotypes through clonal expansion [14]. Based on a simpler 3-locus typing system, *Ff* appears to be even more diverse than *Fk* [12]. However, research on *Ff* has not been conducted to assess whether it possesses a dominant expanding clone complex associated with human infections.

Key questions raised by our current level of knowledge regarding *Fk* and *Ff* derived from human infections and other environments include the following: (1) Is *Fk* 2-d disproportionately represented in STEF, as it is in human fusarioses and in plumbing systems? (2) If so, were the introduction and subsequent establishment of *Fk* 2-d in sea turtle nests due to anthropogenic influence? (3) Or does STEF-associated *Fk* diversity reflect a pattern we currently infer in *Ff*, in which diverse local genotypes cause opportunistic infections? And (4) do *Fk* and *Ff* isolated from sandy beach environments reflect an endemic source or migrant populations with regard to disease occurrence?

The answers to these questions should provide a framework for designing management strategies. Because of bottlenecks and clonal selection, novel pathogens of sea turtle eggs are expected to exhibit reduced allelic variation and increased association among loci when compared with non-STEF-associated populations (i.e., from sink drains, human infections, hatched turtle eggshells, beach sand). If they are endemic, we predict that isolates from diseased sea turtle eggs and non-STEF-associated sources will not exhibit population subdivision [6]. More structured sampling of the built environment and intertidal ecosystems is necessary to elucidate the global population structure and genetic diversity of *Fk* and *Ff*.

As fungal diseases of wildlife become more common, it is imperative that the biological and ecological factors that contribute to the emergence and severity of outbreaks be identified. Establishing whether a pathogen is endemic or novel is an essential first step toward informed management and control of a newly emergent fungal disease [6]. Population genetics provides a key framework for addressing this question. We currently have assembled large collections of *Fk* and *Ff* from the built environment and clinical sources to which STEF-associated isolates can be compared. In addition, a validated sequence-based typing system is available for rapid genotyping and assessment of population structure. These tools, combined with more intensive sampling of sea turtle nesting sites for *Fusarium* species, are key to understanding the current and future impact of STEF worldwide.
